# Building sophisticated sensors of extracellular cues that enable mammalian cells to work as “doctors” in the body

**DOI:** 10.1007/s00018-020-03486-y

**Published:** 2020-03-17

**Authors:** Ryosuke Kojima, Dominque Aubel, Martin Fussenegger

**Affiliations:** 1 Graduate School of Medicine, The University of Tokyo, 7-3-1 Hongo, Bunkyo-ku, Tokyo, 113-0033 Japan; 2 PRESTO, Japan Science and Technology Agency (JST), 4-1-8 Honcho, Kawaguchi, Saitama 332-0012 Japan; 3 IUTA Département Génie Biologique, Université Claude Bernard Lyon 1, Boulevard du 11 Novembre 1918, 69622 Villeurbanne Cedex, France; 4 Department of Biosystems Science and Engineering (D-BSSE), ETH Zurich, Mattenstrasse 26, 4058 Basel, Switzerland; 5 Faculty of Science, University of Basel, Mattenstrasse 26, 4058 Basel, Switzerland

**Keywords:** Synthetic biology, Theranostic cells, Cell-based sensor, Bioengineering, Engineered receptors, Transgene expression

## Abstract

Mammalian cells are inherently capable of sensing extracellular environmental signals and activating complex biological functions on demand. Advances in synthetic biology have made it possible to install additional capabilities, which can allow cells to sense the presence of custom biological molecules and provide defined outputs on demand. When implanted/infused in patients, such engineered cells can work as intrabody “doctors” that diagnose disease states and produce and deliver therapeutic molecules when and where necessary. The key to construction of such theranostic cells is the development of a range of sensor systems for detecting various extracellular environmental cues that can be rewired to custom outputs. In this review, we introduce the state-of-art engineering principles utilized in the design of sensor systems to detect soluble factors and also to detect specific cell contact, and we discuss their potential role in treating intractable diseases by delivering appropriate therapeutic functions on demand. We also discuss the challenges facing these emerging technologies.

## Introduction

When we think we are ill, we go to a clinic or hospital to see a doctor, and the doctor makes a diagnosis and prescribes drugs if necessary. We could say that the doctor “senses” what is happening in the patient, and “responds” to the state of the patients’ symptoms. However, diagnosis can be difficult, and might be too late for a therapeutic agent to be administered at the right time and dose [1]. Therefore, there is enormous interest in so-called theranostic systems [2–5], which are implantable, integrated systems that can automatically diagnose the patients’ disease status and provide appropriate treatment as necessary. An example of such systems would be an electronic device that provides automated closed-loop control of diabetes by continuously monitoring blood-glucose level and delivering the appropriate amount of insulin [6]. However, it is difficult to develop electronic sensor systems that can sense the presence of various disease biomarkers with current technologies. On the other hand, mammalian cells are inherently capable of sensing extracellular signals and responding to them; further, they can produce output bioactive molecules continuously as long as they are supplied with sufficient nutrients and energy. Due to recent advances in synthetic biology, an interdisciplinary area between biology and engineering [7, 8], it has become possible to design custom sensing abilities and build them into mammalian cells, enabling the engineered cells to sense the presence of various disease markers, activate intracellular signaling, and exert therapeutic functions (e.g., secretion of therapeutic proteins) on demand. Intrabody implantation/infusion of such engineered cells allows continuous monitoring of disease states and on-demand production and delivery of therapeutic molecules, just as if “doctors” were at work within the body.

The key to constructing such systems is to endow mammalian cells with the required custom input-sensing abilities. In this review, we introduce the state-of-art engineering principles utilized in the design of various sensor molecules to detect both soluble factors and specific cell contact. For sensing soluble factors, we introduce examples of sensor systems using both natural (or existing) receptors (including those with slight modifications), and synthetic receptors developed via bottom-up approaches. For sensing specific contact, we introduce the principles of receptor engineering of immune cells, as well as some unconventional sensor development strategies that harness the biophysical movement of rationally designed chimeric proteins for engineering non-immune cells. We also discuss the challenges that face these emerging technologies.

## Sensing soluble factors

Soluble factors (both proteins and small molecules) are the most easily accessible disease markers. By rewiring the downstream signaling of either natural or synthetic receptors to transgene expression, we can engineer mammalian cells to sense the presence of disease markers and provide therapeutic functions on demand. Generally, engineered cells for sensing soluble factors can be encapsulated in immuno-isolative microcapsules (e.g., alginate beads [9]) or a macro encapsulator [10] such that soluble disease markers, secreted effector proteins, and nutrients for the cells can permeate through the shell of the implant while the cells are protected from host immune-response systems.

### Harnessing the power of natural receptors

The downstream signaling from natural receptors that bind to various biological molecules can be rewired, either by using the natural downstream signaling of the receptor or by fusing some effector module to trigger target transgene expression, so that the cells can sense various soluble disease markers and secrete therapeutic proteins when necessary. One class of receptors that has been used in this way is the G protein-coupled receptors (GPCRs); these receptors contain multiple transmembrane segments that can detect molecules outside the cells and activate downstream signal transduction through coupled G proteins. If a GPCR that can sense a target molecule/stimulus is available, it can be ectopically expressed to force cells to respond.

**Fig. 1:**
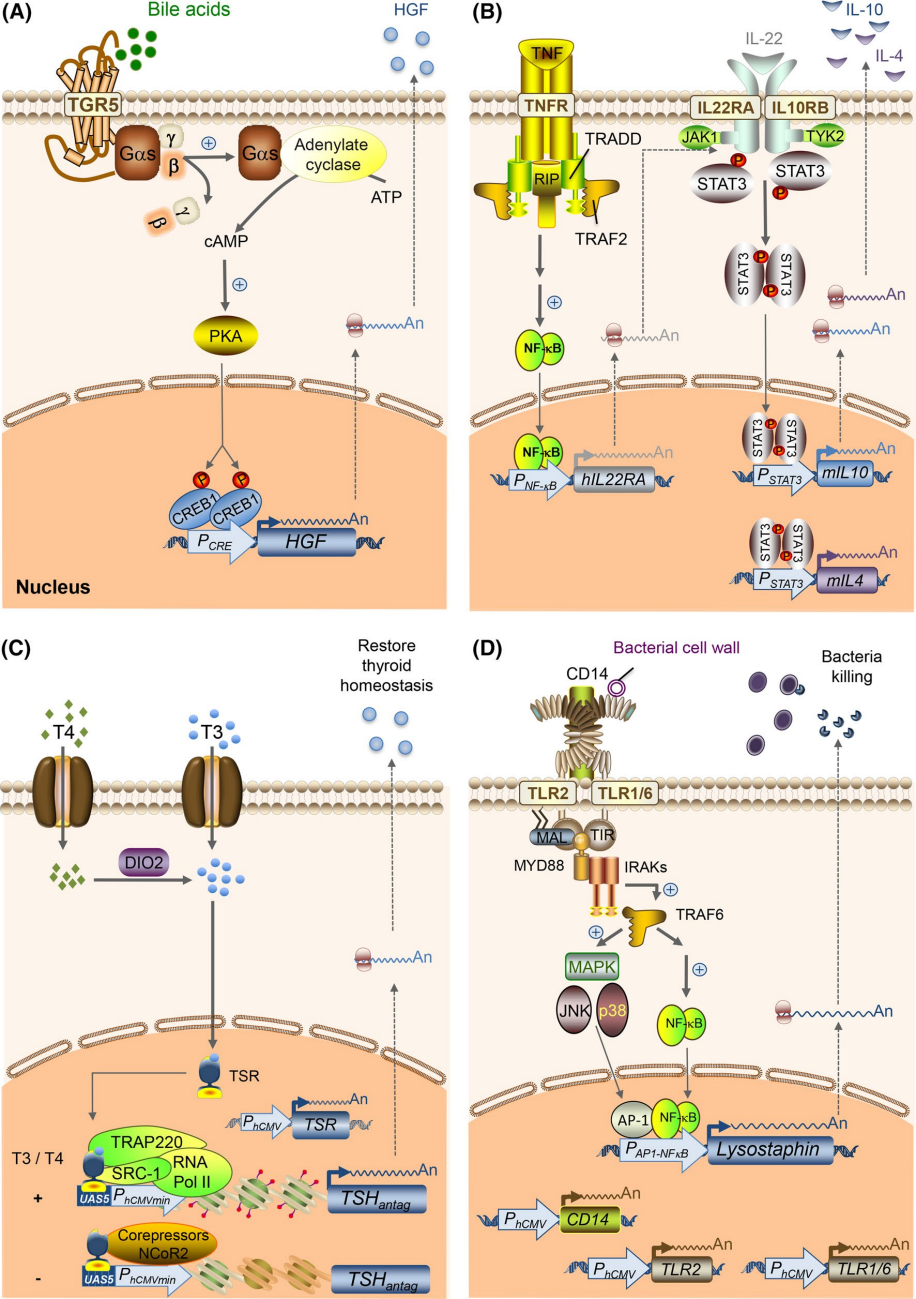
Harnessing the power of natural/existing receptors to enable mammalian cells to sense various soluble disease markers. **a** Use of GPCR (G protein-coupled receptor) for bile acid sensing. A GPCR to sense bile acid (TGR5) senses extracellular bile acids and triggers Gαs-mediated activation of adenylate cyclase. This converts intracellular ATP (adenosine triphosphate) to cAMP (cyclic adenosine monophosphate), which binds to the regulatory subunits of protein kinase A (PKA). The catalytic subunits of PKA translocate into the nucleus and phosphorylate cAMP-responsive binding protein 1 (CREB1). Phosphorylated CREB1 triggers transgene expression from synthetic promoter P_CRE_ engineered to contain different CREB1 response elements (CRE). When HGF is used as an output in this inducible gene expression system, the whole system works as a liver-protection device. **b** Use of cytokine receptors for sensing TNF (tumor necrosis factor) and IL22 (interleukin 22) levels associated with psoriasis. TNF binds to endogenous or ectopically expressed TNF receptor, which leads to NFκB (nuclear factor kappa B)-triggered expression of hIL22RA (IL22 receptor subunit alpha-1). In the presence of IL22, hIL22RA heterodimerizes with endogenous hIL10RB (IL10 receptor subunit beta), which triggers the corresponding JAK/STAT (Janus kinase/signal transducers and activators of transcription) signaling cascade. Phosphorylated STAT3 dimerizes and translocates to the nucleus and activates the expression of murine IL4 and IL10 triggered by STAT3 promoter. Secreted IL4 and IL10 exert anti-inflammatory effects to treat psoriasis. Since the whole system works only when both TNF and IL22 are present, it can be said that the signaling cascades provide AND-gate expression logic. **c** Use of nuclear receptor for sensing thyroid hormone levels. The thyroid hormone-responsive gene switch (TSR) comprises the ligand-binding domain of the thyroid receptor (TR) connected to the DNA-binding domain of Gal4, which binds to a Gal4-specific operator sequence linked to a minimal promoter. In the absence of the thyroid hormones T3 and T4, TSR associates with corepressors, such as nuclear receptor corepressor 2 (NcoR2), which inhibits gene repression. In the presence of T3 and T4 (converted to T3 by DIO2), TSR interacts with coactivators, such as steroid receptor coactivator-1 (SRC-1) and thyroid hormone receptor-associated protein complex 220-kDa component (TRAP 220), which triggers transgene expression. When thyroid-stimulating hormone receptor (TSHR) antagonist (TSH_antag_) is used as an output, the system constantly monitors systemic T3 level and express TSH_antag_ to neutralize excessive activation of TSHR by autoantibody, as seen in Graves disease, restoring thyroid hormone homeostasis through a synthetic negative feedback loop. **d** Use of toll-like receptors (TLRs) for sensing bacteria. Constitutively expressed human TLR2, TLR1, TLR6, and the CD14 co-receptor recognize components of bacterial cell walls. Upon stimulation, TLR2 associates with Mal (MYD88-adapter-like) and MYD88 (myeloid differentiation primary response 88), and the subsequent formation of a complex of IRAKs (interleukin-1 receptor-associated kinases) and TRAF6 (TNF receptor-associated factor 6) is induced. TRAF6 then undergoes phosphorylation and ubiquitylation, which trigger downstream translocation of AP-1 (activator protein 1) and NF-kB into the nucleus. These transcription factors initiate the expression of a transgene. When lysostaphin, a bacteriolytic enzyme highly lethal to *Staphylococcus aureus,* is used as an output, this system is capable of killing even MRSA (methicillin-resistant *S. aureus*)

For example, Rössger et al. ectopically expressed human dopamine receptor D1 (DRD1) on mammalian cells, and rewired its native signaling cascade, involving G_αs_, cAMP (cyclic adenosine monophosphate), PKA (protein kinase A), and CREB (cAMP-responsive binding protein) [11] (Fig. 1a). By transfecting an expression cassette for DRD1 together with a transgene expression cassette bearing a synthetic promoter containing CREB1-specific cAMP response elements (CRE), they enabled the cells to sense the environmental dopamine level and express an output protein accordingly. When engineered cells capable of dopamine-inducible ANP (atrial natriuretic peptide, a powerful vasodilator attenuating high blood pressure) expression were implanted into hypertensive mice, the cells produced ANP when the reward system of the mice was stimulated by food, sexual arousal or an addictive drug, thereby reducing the blood pressure of the mice to the normal physiological level. In this context, the cells indeed work as intra-body “doctors” which can “diagnose” the target abnormal state and “prescribe” medicine accordingly.

As another example using GPCR as a sensor, Bai et al. developed designer cells that sense bile acid levels associated with liver injury and secrete hepatocyte growth factor (HGF) to protect the liver [12]. They ectopically expressed human GPCR TGR5, which senses bile acid, in mammalian cells. Activation of this receptor triggers G_α6_-mediated activation of adenylate cyclase, which converts ATP (adenosine triphosphate) to cAMP. The cAMP surge was rewired to expression of HGF induced by an inducible promoter containing cAMP-response elements (CRE). With this setup, HGF expression is induced in response to an increase of blood bile-acid level triggered by liver injury.

These are examples of “closed-loop” theranostic cell-based devices using GPCR that have the ability to sense a disease state and reverse it via a feedback mechanism. By changing the sensor module, it is possible to construct different kinds of systems responding to various biological molecules. For instance, Liu et al. reported a sensitive free-acid-regulated transgene switch in mammalian cells by using GPR40 (G protein-coupled receptor 40) as a fatty acid sensor [13]. Sedlmayer et al. used human formyl peptide receptor 1 (FPR1) to sense formyl peptides secreted by bacteria, and combined it with an NFAT (nuclear factor of activated T cells)-inducible expression cassette of LuxS (S-ribosylhomocysteine lyase), which generates AI-2 (autoinducer-2), a universal modulator of bacterial quorum-sensing behaviour [14]. This transgene expression device may provide opportunities for future anti-infective strategies.

By using GPCRs that can sense external stimuli, it is also possible to precisely control output expression voluntarily. For example, by using cTAAR1, a GPCR capable of sensing guanabenz (a clinically licensed antihypertensive drug) as a sensor and GLP-1 again as an output, it was shown to be possible to control blood sugar level by taking a drug used to treat a disease associated with diabetes [15]. By using the concentration of a drug used to treat one aspect of a metabolic disease state (in this case, hypertension) as a trigger to secrete a therapeutic protein that acts against another aspect of the disease state, such as obesity or hyperglycemia, it would become possible to simultaneously treat multiple pathologies. Likewise, when melanopsin, a GPCR capable of sensing blue light, and GLP-1, a peptide hormone that promotes insulin release from beta cells, are used as a sensor and an output, respectively, cells can control blood sugar level according to the intensity of light shone on the animal [16] (although this system does not sense a soluble marker, we mention it here since it involves GPCR engineering).

Another interesting application of GPCR as a sensor is to synthetically control cellular motility. Park et al. showed that expression of a Gi-coupled engineered GPCR (RASSL) can program cells to sense the gradient of the GPCR ligand and migrate to the source of the ligand [17]. This technology would be useful for enhancing the efficacy of T-cell-based cancer therapy (see below). (Note that RASSL is not a natural receptor, but again, we include it here in the context of GPCR sensors.)

Not only GPCRs, but also other types of proteins can be used to construct sensors for different inputs. For example, cytokine receptors can be used for sensing the state of disease associated with immune system disorders. Schukur et al. used TNF (tumor necrosis factor) receptor and IL22 (interleukin 22) receptor to sense the state of psoriasis [18] (Fig. 1b). Constitutive expression of TNF receptor (TNFR) on mammalian cells enables the cells to first sense the presence of TNF, and this triggers expression of hIL22RA (human IL22 receptor subunit alpha) driven by inducible promoter responsive to NFκB (nuclear factor-kappa B). The expressed hIL22RA is thought to heterodimerize with endogenous IL10RB (IL10 receptor subunit beta), and this complex senses the presence of IL22, driving expression of output molecules to treat psoriasis, IL4 and IL10, via STAT3 (signal transducer and activator of transcription 3) signaling. Thus, this “cytokine converter” exerts its function only when both TNF and IL22 coexist (AND-gate logic), which is a characteristic of psoriasis.

Nuclear receptors can be also used for sensing diseases. For sensing fatty acid level, Rössger et al. constructed a fusion protein comprising human PPARα (peroxisome proliferator-activated receptor alpha) and TtgR (phloretin-responsive repressor), which binds to a TtgR specific operator linked to a minimal promoter [19]. In the absence of fatty acids, this fusion protein associates with an endogenous inhibitory complex to repress transgene expression, while an endogenous activation complex is recruited when the fatty acid level rises. Since PPARα and TtgR are fused, the recruited activation complex is brought close to the minimal promoter, thereby inducing the downstream transgene expression. By using pramlintide, a hormone that suppresses appetite, as an output, cells can remotely suppress the appetite of mice when the blood fatty acid level exceeds the normal range, which could be potentially useful for treating metabolic disorder.

Another example of a nuclear receptor-based sensor is thyroid receptor. Saxena et al. reported designer cells bearing a closed-loop gene circuit that controls thyroid hormone homeostasis as a candidate for treating Graves’ disease [20] (Fig. 1c). As a thyroid-sensing receptor (TSR), they used the ligand-binding domain of human TRα (thyroid receptor alpha) linked to the DNA-binding domain of yeast Gal4 protein, which binds to a Gal4-specific operator sequence linked to a minimal promoter. TSR associates with co-repressors consisting of multiple proteins including NCoR2 (nuclear receptor corepressor 2) and SMRT (silencing mediator for retinoid or thyroid hormone receptor) in the absence of thyroid hormones T3 and T4; in this case, transgene expression under the inducible promoter is not activated. In the presence of T3 and T4, TSR associates with co-activators such as SRC-1 (steroid receptor coactivator-1) and TRAP220 (thyroid hormone receptor-associated protein complex 220-kDa component) that mediate gene expression. Since Graves’ disease is an autoimmune disorder associated with hyperthyroidism due to autoantibodies that bind to the thyroid-stimulating hormone receptor (TSHR) and trigger thyroid hormone release at the thyroid gland, Saxena et al. selected a TSHR antagonist as an output. The implanted cells bearing the closed-loop gene circuit secrete the TSHR antagonist when blood thyroid level is upregulated, and thereby restore the hypothalamus–pituitary–thyroid axis to euthyroid hormone levels.

Toll-like receptors (TLRs) can be also used for sensing diseases, especially infection. Liu et al. developed immunomimetic cells that protect mice from MRSA (methicillin-resistant *Staphylococcus aureus*) infection [21] (Fig. 1d). They co-expressed human TLR2, TLR1/6 and CD14 on HEK-293 cells and rewired its downstream signaling mediated by NF-κB (nuclear factor-kappa B) via a synthetic inducible promoter bearing carefully tuned AP-1 (activator protein 1) and NF-κB responsive elements, enabling the cells to sense the presence of extracellular microbial components such as bacterial cell wall. They linked expression of a bacteriolytic enzyme highly lethal to *S. aureus* to this device and showed that implanted cells bearing this closed-loop gene network can completely cure even late acute MRSA infection.

Another class of natural receptors available for engineering cellular function is ion channels. Xie et al. used ectopic expression of a voltage-gated calcium channel (Ca_v_1.3) on HEK-293 cells to transform the cells into β-cell-mimetic designer cells [22]. They showed that expression of this channel is decisive for glucose sensing in non-endocrine human cell types. When the engineered cells sense a high blood glucose level, this leads to upregulated glucose uptake via Glut1 (glucose transporter 1), increased ATP production, closure of ATP-sensitive potassium channels, and Ca_v_1.3-mediated Ca^2+^ influx. By encoding an output gene for GLP-1 (glucagon-like peptide-1) under an NFAT (nuclear factor of activated T cells)-dependent inducible promoter and implanting the engineered cells into a mouse model of diabetes, they obtained a closed-loop system to correct hyperglycemia in vivo. Bai et al. reported another example in which an ion channel is harnessed to endow cells with an additional function; they used TRPM8 (transient receptor potential 8), which is stimulated by menthol or exposure to a cool environment [23]. When TRPM8 is expressed on HEK-293 cells, activation of this channel allows Ca^2+^ to pass through the plasma membrane, thereby increasing the intracellular Ca^2+^ level. Again, this intracellular Ca^2+^ upregulation can be rewired to transgene expression driven by an NFAT-dependent inducible promoter. When modified insulin or mActRIIB^ECD^-hFc (a modified, activin type IIB, receptor ligand trap protein) was used as an output, the system could alleviate hyperglycemia in a mouse model of type 1 diabetes or reverse muscle atrophy in a model of muscle wasting, respectively.

By conjugating an ion channel to a functional protein, it is also possible to make mammalian cells capable of sensing radio waves [24]. For this purpose, GFP-tagged ferritin containing iron oxide is expressed intracellularly, and associates with a fusion protein of TRPV1 (Transient Receptor Potential Vanilloid 1, a temperature-sensitive GPCR) and anti-GFP nanobody. When cells are irradiated with radio waves, this input is transduced into channel activation, and the subsequent calcium upregulation can be rewired to transgene expression.

Thus, by harnessing the power of natural receptors to sense various molecules and carefully rewiring their downstream signaling, one can program mammalian cells to sense a wide range of extracellular cues and provide various output functions in response.

### Building synthetic chimeric receptors to expand the repertoire of detectable soluble molecules

When no receptor is available for sensing soluble molecules of interest, it is also possible to build up fully customized synthetic receptors by means of bottom-up protein engineering.

**Fig. 2:**
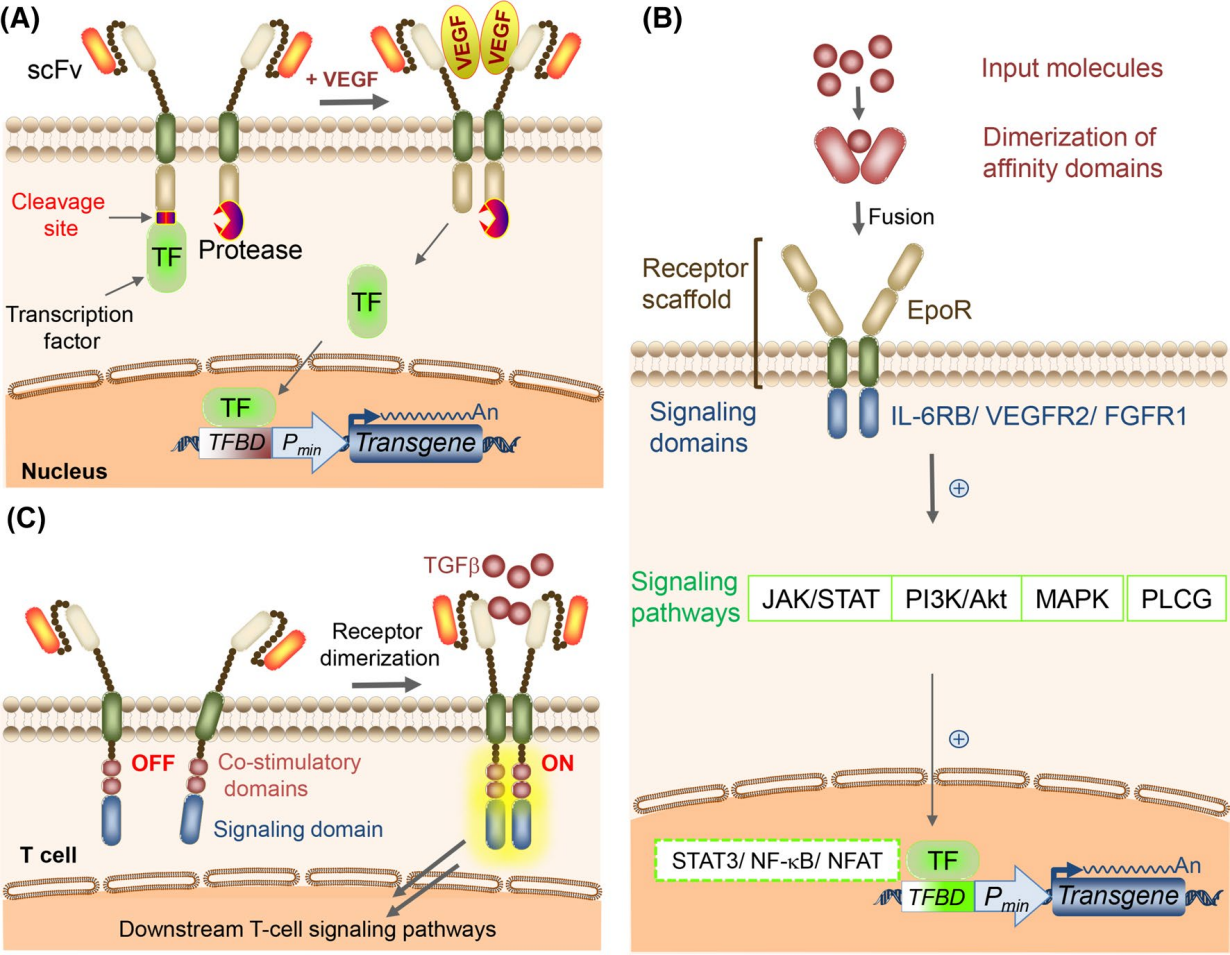
Soluble protein sensors built by means of bottom-up approaches. **a** VEGF (vascular endothelial growth factor) sensor based on MESA (modular extracellular sensor architecture). Binding of dimeric VEGF induces dimerization of the hetero receptors, which results in *trans*-cleavage and release of a transcription factor (TF) from the plasma membrane. TF binds to the transcription factor-binding domain (TFBD) and induces transgene expression. **b** GEMS (generalized extracellular sensor) platform to sense various molecules. The affinity domains connected to the Epo receptor (EPOR) scaffold dimerize upon addition of input molecules. The affinity domains can be various proteins including, but not limited to, FKBP and FRB (for sensing rapamycin), camelid heavy chain antibody A52 (for sensing an azo dye RR120), heavy chain and light chain of the variable chain of nicotine antibody (for sensing nicotine, fused separately in the receptor for heterodimeric receptors based on nicotine-induced stabilization of the heavy and light chain interaction), and two different scFvs to distinct epitopes of prostate-specific antigen (PSA). Upon dimerization of the extracellular domain, the orientation of the intracellular domain changes, triggering downstream signaling pathways. Different kinds of intracellular domains, such as those of IL-6RB, VEGFR2, and FGR1, can be used to activate corresponding downstream signaling pathways (JAK-STAT pathway, PI3K/Akt (phosphatidylinositol 3-kinase/protein kinase B; induced by VEGFR2) pathway, PLCG (phospholipase C gamma) pathway, MAPK (mitogen-activated protein kinase) pathway), which can be rewired to transgene expression from inducible promoters. **c** Chimeric antigen receptor (CAR) engineered to sense soluble TGF-β (tumor growth factor-β). CAR contains an extracellular scFv (single-chain variable fragment) that binds to TGF-β and is linked to the CD28 and CD3ζ endodomains. Upon sensing dimeric soluble TGF-β, CAR dimerizes, and downstream T-cell signaling is triggered

Schwarz et al. reported a synthetic receptor system to sense a soluble protein based on “modular extracellular sensor architecture” (MESA) [25] that uses dimerization-induced cleavage of a transcription factor [26] (Fig. 2a). They conjugated a single chain antibody (scFv) against VEGF (vascular endothelial growth factor) to a MESA scaffold (containing CD28 transmembrane domain) whose intracellular domain is either TEV protease or a synthetic transcription factor, tTA, fused with a TEV cleavage site. Since VEGF works as a homodimer, the presence of VEGF induces dimerization of the receptor, causing TEV to encounter its cleavage site. This releases the membrane-tethered tTA, and the released tTA translocates into the nucleus to trigger transgene expression. The transcription factor can be exchanged to trigger other outputs. By fusing the dCas9-transcription factor conjugate, they also showed it is possible to control endogenous gene expression with this device when co-expressed with its guide RNA (dCas9 is a mutant of Cas9 whose endonuclease activity has been removed; for details of the CRISPR/Cas9 system, see Ref. [27]). By conjugating a transcription factor (such as VP64) and using it simultaneously with gRNA to target specific loci, it is possible to selectively upregulate transcription of target gene.

Similarly, Baeumler et al. reported split dCas9-based receptors capable of coupling biologically relevant input signals to activate transgene expression [28]. They split the dCas9-transactivator (VP64) into two parts: dCas9(N) and dCas9(C)-VP64, and conjugated each component to proteins that dimerize, but are cleaved by TEV protease after the input molecule binds to its receptor. For example, they conjugated TEV(N) (one part of split TEV) and dCas9(N) to VEGFR-2 via a TEV cleavage site (TCS) (VEGFR2 (extracellular domain-transmembrane domain)-TEV(N)-TCS-dCas9(N)), and conjugated TEV(C) (the other part of split TEV) and dCas9(C)-VP64 to VEGFR1 via TCS (VEGFR1 (extracellular domain-transmembrane domain)-TEV(C)-dCas9(C)-VP64). These receptors heterodimerize upon binding of VEGF to the receptor. This induces reconstitution of TEV and dCas9-VP64, and dCas9-VP64 is cleaved from the plasma membrane by TEV, thereby triggering expression of the target gene (determined by the co-expressed gRNA). They also showed that this kind of TEV-based framework is applicable to various types of receptor. For example, by conjugating each part of split dCas9-VP64 to GPCR via TCS, and conjugating TEV to β-arrestin, which is recruited to GPCR, TEV is recruited to GPCR upon ligand binding, and each part of the split dCas9-VP64 is cleaved from the membrane. Spontaneously reconstituted dCas9-VP64 can then trigger downstream gene expression.

Scheller et al. reported a receptor scaffold that enables an antibody-specific soluble molecular input to activate JAK/STAT (Janus kinase/signal transducer and activator of transcription), MAPK (mitogen-activated protein kinase), PLCG (phospholipase C gamma) or PI3K/Akt (phosphatidylinositol 3-kinase/protein kinase B; induced by VEGFR2) signaling, which is rewired to transgene expression [29] (Fig. 2b). They used Epo receptor (EpoR) as a scaffold of the sensor proteins and extracellularly conjugated target-affinity domains that dimerize upon binding of input molecules. Also, depending on the intracellular domain conjugated to the scaffold, it is possible to trigger different downstream signaling pathways (IL-6RB to trigger the JAK/STAT pathway, VEGFR2 to trigger the PI3K/Akt or PLCG pathway, FGFR1 to trigger the MAPK pathway). By co-transfecting an output expression cassette that responds to activation of each signaling pathway, various proteins (e.g., therapeutic proteins) could be expressed in response to input proteins such as disease markers. The success of this platform, called generalized extracellular molecule sensors (GEMS), shows that cellular receptor components are quite modular, and we can rationally engineer synthetic receptors that can sense various types of input molecules.

As an alternative approach to sense soluble input molecules, Chang et al. rewired T-cell responses to soluble factors with chimeric antigen receptors (CAR) [30] (Fig. 2c). As discussed later, chimeric antigen receptor is usually used for engineering T cells to respond to a specific antigen expressed on the membrane of target cells. However, Chang et al. showed that ligand-mediated CAR dimerization can actually trigger a T-cell response to soluble ligands, and the responsiveness can be fine-tuned by adjusting the mechanical coupling between the CAR’s ligand bindings, as well as the signaling domains. By using dimeric proteins as inputs, or by using two CARs that simultaneously bind a monomeric input molecule at two different epitopes, they showed that it is possible to make the system capable of sensing various kinds of molecules, including GFP, TGF-β (transforming growth factor beta), and CD19ecto. By using TGF-β CAR, they were able to convert immunosuppressive TGF-β to a stimulant for T cells. This should enable conversion of a potent immunosuppressive cytokine associated with solid-tumor microenvironments to provide a more immuno-stimulating environment.

Thus, we can build customized synthetic receptors sensing various soluble ligands by using receptor protein dimerization in response to ligand binding as a driving force to trigger the target downstream signaling.

## Sensing cellular contact

So far, we have described sensor systems for soluble molecules, but certain types of cell-based therapies require sensors of specific cell contact via membrane proteins. For example, when we try to engineer cells that can sense the presence of cancer cells and kill them specifically, the engineered cells must be able to sense a specific antigen expressed on cancer cells and exert a cell-killing function. In this section, we review sensors of specific cell contact. It should be noted that patient-derived cells must be exclusively engineered for real clinical applications, because we cannot use immuno-isolative encapsulation systems (in contrast to the case of cells for sensing soluble molecules). Although most of the systems introduced here are still at a pre-clinical, proof-of-concept stage, it should be possible to apply many of these engineering principles to patient-derived cells in the future.

### Engineered receptors for sensing cell contact functional in immune cells

**Fig. 3:**
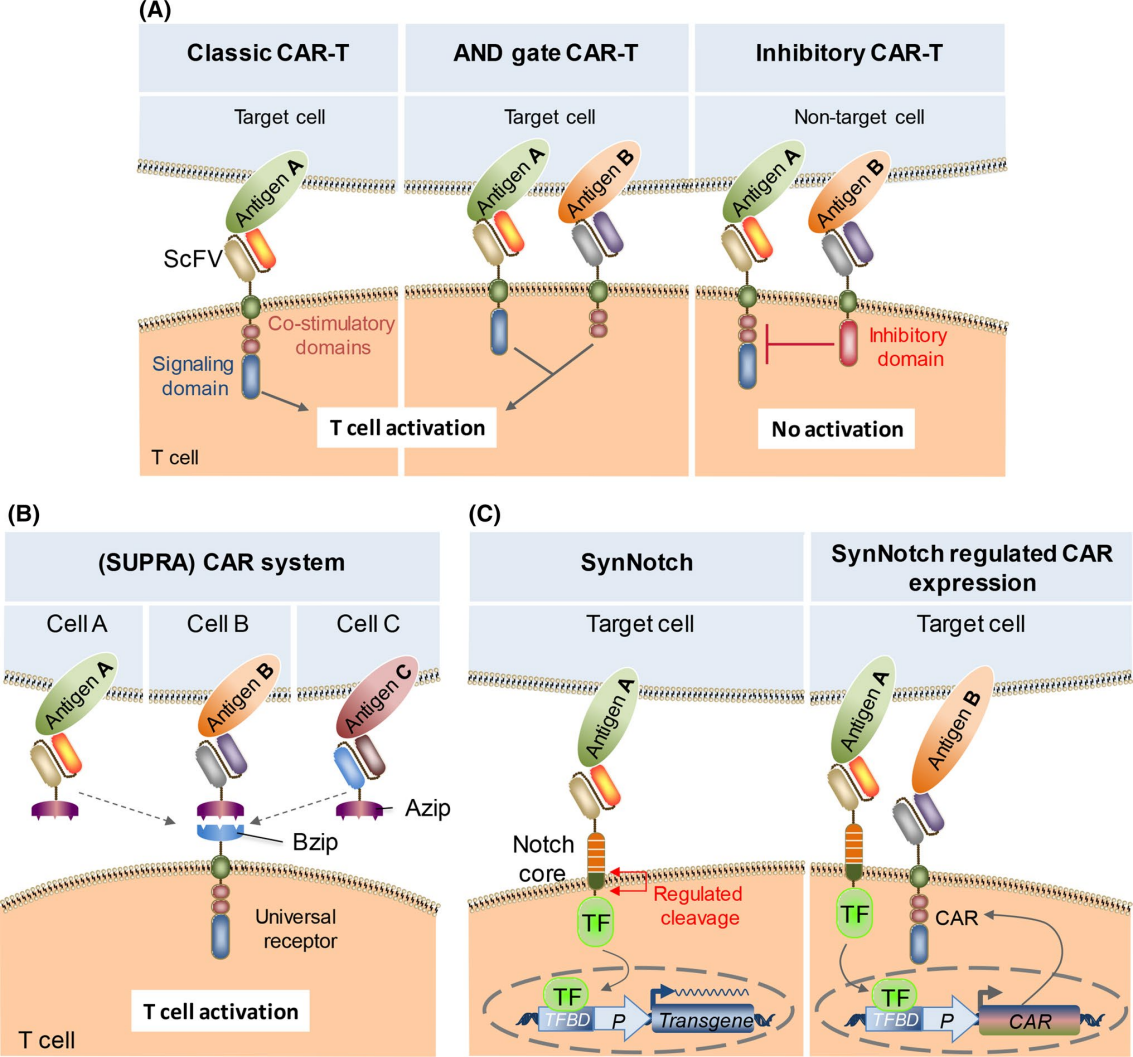
Engineering immune cells to sense specific cell contact. **a** Boolean logic gates made with CAR. Classic CAR consists of an extracellular scFv that binds to a target antigen, a transmembrane domain, co-stimulatory domain, and signaling domains. The domains used for CAR are different in different generations of CARs. Expression of CAR enhances the target-cell-killing activity of T cells. In order to construct an AND gate CAR-T, CAR bearing an inefficient scFv against antigen A is fused to the intracellular signaling domain, and another receptor bearing an efficient scFv against antigen B is fused to the intracellular co-stimulatory domain. Co-expression of these two receptors enables activation of downstream signaling only when the cell in contact expresses both antigen A and antigen B. In order to construct an A AND NOT B logic with CAR, co-receptor comprising scFv against antigen B and an intracellular inhibitory domain (from CTLA-4 (cytotoxic T lymphocyte-associated antigen 4), or PD-1 (programmed cell death protein 1) is co-expressed with CAR sensing antigen A. **b** SUPRA CAR system that enables multiplexed and logical control of T cell response by the use of universal receptor and soluble antigen-recognizing protein. This system is composed of a ZipCAR that has a leucine zipper (BZip) as the extracellular domain, and zipFv that has a scFv against target antigen fused to a cognate leucine zipper (AZip) capable of binding to BZip. By changing the scFv of zipFv, the target antigen can be easily changed with the same T cells bearing a universal receptor. **c** SynNotch system that endow cells with custom cell-sensing ability. As long as the Notch core is preserved, its extracellular domain and intracellular domain can be swapped with a binder directed against the target antigen (e.g., scFv) and a custom transcription factor, while retaining the feature that the intracellular domain is cleaved in a regulated manner upon target cell recognition. Release of transcription factor from the plasma membrane can be rewired to transgene expression. By encoding specific cytokines as a transgene, it is possible to produce a la carte cytokine profiles. By using CAR against a different antigen as an output of the SynNotch system, it is also possible to form AND gate logic in T cells to sense specific target cells

The best-known cell-contact sensor is the chimeric antigen receptor (CAR) for T cells. CAR consists of an extracellular scFv domain to recognize the target antigen, a transmembrane domain, and an intracellular domain to trigger downstream T cell signaling [31]. CAR endows T cells with the new ability to target a specific antigen, by rewiring a custom input to downstream T cell signaling pathways (Fig. 3a). CAR-T cells targeting CD19 have already been approved by the FDA (U.S. Food and Drug Administration), as well as regulatory authorities in many other countries, to treat several types of leukemia, and are on sale as Tisagenlecleucel (Kymriah) from Novartis, and Axicabtagene Ciloleucel (Yescarta) from Kite Pharma/Gilead. Intensive research aimed at improving the system and diversifying the targets of CAR is continuing at both the clinical and preclinical level [32].

In order to increase the efficacy of the CAR system, researchers have been focusing on engineering its intracellular domain, and there are already several generations of CARs [31]. The first-generation CAR contains only CD3ζ (or FcRγ) as an intracellular domain, but it has been realized that installing multiple signaling domains enhances the efficacy of CAR. In second-generation CAR, a single stimulatory domain (either from CD28 or 4-1BB) was added between the transmembrane domain and intracellular domain of CD3ζ to enhance downstream signaling (Kymriah and Yescarta use second-generation CARs). In third-generation CAR, two co-stimulatory domains are installed to further enhance downstream signaling.

By engineering CAR-T cells to secrete additional effector molecules, their activity can be enhanced even further. For example, CAR-T cells bearing an inducible expression cassette of an immune-stimulatory cytokine such as IL12 have been reported as TRUCKs (also called fourth-generation CARs) that are capable of more efficiently treating cancers by stimulating surrounding immune cells [33]. More recently, CAR-T cells capable of secreting BiTE (bispecific T-cell engager) that can recruit untransduced bystander T cells against cancer were reported [34]. Thus, equipping CAR-T cells with additional functions is a promising strategy to enhance the efficacy of CAR systems.

Another important approach for improving CAR would be to increase the specificity. From this viewpoint, there are several approaches, using an AND gate or an A AND NOT B gate with different types of signaling domains (Fig. 3a). AND-gate CAR exerts its function only when the cells sense the presence of both antigen A and antigen B. This functionality was constructed by using two different CARs; one comprising an inefficient scFv and signaling domain without a co-stimulation domain, and the other comprising an efficient scFv and a co-stimulation domain without a signaling domain [35]. Only when the target cell expresses both antigens can the whole system trigger downstream signaling. On the other hand, the A AND NOT B type exerts its function when the target cell expresses antigen A, but downstream signaling is shut off when antigen B is also expressed. This functionality was again constructed by using two different chimeric receptors [36]. The receptor for antigen A is a normal CAR, whereas for sensing antigen B, an inhibitory domain from CTLA-4 or PD1 is used as the intracellular domain to form an inhibitory CAR. With these kinds of logic operations, it is possible to selectively define target cells based on the combined expression levels of multiple biomarkers, even if there are no specific biomarkers on the target cells.

For construction of the above logic gates, researchers split the system functions into two different receptors. Also, by splitting a single CAR into different parts in different ways, it becomes possible to rationally control CAR functions in other ways. Cho et al. split the extracellular domain of a CAR (scFv) from the remaining part (transmembrane domain and intracellular domain) and conjugated a leucine zipper to each part [37] (Fig. 3b). This allowed for reassembly of scFv and the remaining CAR part in situ through the interaction of the leucine zippers. This design allows for fine-tuning of the degree of T cell activation through multiple control points of T cell activation, such as the binding affinity of the leucine zippers, the possibility of adding competing leucine zippers, and the binding affinity of the scFv. The most important feature of this system is that it is not necessary to re-engineer T cells for adapting to different target antigens, since one can simply use a different scFv, depending on the target antigen. This approach would substantially reduce the cost and time requirement for expanding the target range of CAR-T cells.

So far, we have introduced various ways to engineer immune cells with CAR, but there is another class of synthetic receptor that can endow cells with custom input/output functions; a synthetic Notch-based receptor called synNotch. Notch receptor is a single-pass transmembrane protein that plays important roles with Delta in juxtacrine cell-to-cell communication. When Notch binds to Delta in the process of cell–cell contact, the intracellular domain of Notch is cleaved from the plasma membrane, and this domain functions as a transcription factor to drive downstream gene expression. Morsut et al. found that this feature of Notch is retained even if the extracellular domain and the intracellular domain are swapped with a custom antigen binder and an artificial transcription factor, respectively, as long as the Notch core is appropriately preserved [38] (Fig. 3c). When the scFv of synNotch binds to the target antigen, the protease cleavage site of the Notch core is exposed due to mechanical force, initiating a multistep process leading to release of the intracellular transcription factor. If an inducible gene expression cassette that responds to the transcription factor is introduced, cells can express the output protein depending on specific cell contact.

As is apparent from its working principle, synNotch induces only defined gene expression, while CAR activates overall T cell signaling. Leveraging this advantage, Roybal et al. showed that synNotch can drive a la carte cytokine expression in T cells [39]. For example, they showed that T cells can be engineered to specifically secrete an immunosuppressive cytokine (IL10) or an immunostimulatory cytokine (IL2) without producing any other type of cytokine, which is impossible with CAR. They also showed that T cells can be programmed to differentiate into antitumor Th1 fate upon specific cell contact by using synNotch-induced expression of a transcription factor, Tbet.

As CAR and synNotch work completely orthogonally, it is possible to form logic gates by using the two receptors. For example, Roybal et al. described an AND gate CAR output that is triggered only when the target cells express both antigens A and B, by using an expression cassette of CAR against antigen B, whose expression is induced by input to synNotch by antigen A [40] (Fig. 3c). Although they presented a proof of principle in a relatively synthetic setting (with CD19 and GFP as target antigens), a recent study showed that this approach also works in a more biologically relevant setting. Tumor-associated ROR1 is a candidate target for CAR-T, but CAR-T against ROR1 induces lethal bone marrow failure due to recognition of ROR1^+^ stromal cells. To overcome this issue, Srivastava et al. developed AND gate CAR-T cells constructed with synNotch for EpCAM (epithelial cell adhesion molecule, highly expressed in some types of cancers) which induces expression of ROR1 CAR [41]. This enables ROR1 CAR expression selectively within the tumor that expresses both ROR1 and EpCAM, resulting in tumor regression without toxicity.

Thus, various strategies to endow immune cells with custom input/output functions have been developed by receptor engineering.

### Harnessing dynamic movement of proteins for sensing cell contact

**Fig. 4:**
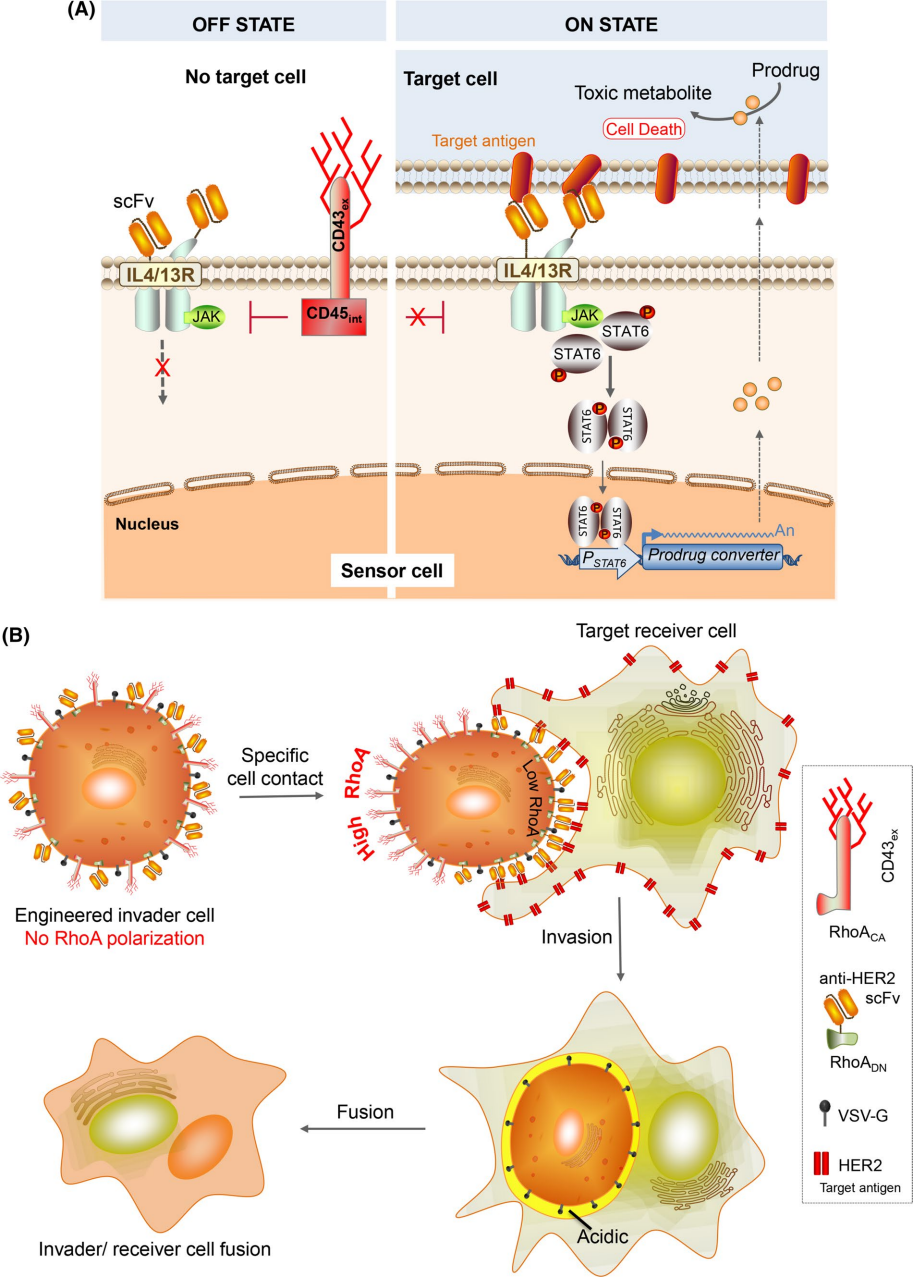
Harnessing dynamic movement of proteins for sensing cell contact. **a** Specific cell-contact sensor based upon physical segregation of CD43ex-45int (conjugate of the extracellular and transmembrane domain of CD43 and the intracellular domain of CD45). Without a target cell, CD43ex-45int lies in the vicinity of the chimeric IL4/13 receptor (IL4/13R), suppressing its downstream JAK-STAT signaling. Once scFvs on IL4/13R bind to the target antigen on a target cell, CD43ex-45int is segregated from cell–cell interface due to the physical force applied to the large extracellular domain of CD43ex-45int. Then, CD43ex-45int can no longer suppress downstream JAK-STAT signaling, which triggers downstream transgene expression induced by a STAT6-responsive promoter. By expressing a cell-penetrating prodrug-converting enzyme as an output, it becomes possible to kill target cells by applying the prodrug. **b** Use of CD43 segregation to program target-specific cell invasion. Constitutively active RhoA is conjugated to CD43ex, and dominant-negative RhoA is conjugated to an antigen-binder. Without a target cell, there is no polarization of RhoA activity in the cells. However, when the engineered cell binds to a target cell via scFv-antigen interaction, CD43ex-RhoA_CA_ is segregated from the cell–cell interface due to the physical force applied to the large extracellular domain of CD43, which induces polarization of RhoA activity, enabling the engineered cell to invade the target cell. Furthermore, expression of fusogenic VSV-G on the invader cell causes fusion of the cytosol of the invader cell and receiver cell, enabling delivery of functional protein into the receiver cell, as well as eventual ablation of the target cell

So far, we have only introduced strategies to endow cells with custom cell-sensing ability by engineering receptors. However, it is also possible to construct specific cell-contact-sensing functions by orchestrating the dynamic movement of multiple membrane proteins. We reported that physical movement of carefully designed membrane proteins upon specific cell contact can control OFF/ON switching of synthetically constructed JAK-STAT signaling [42] (Fig. 4a). We co-expressed target-recognizing chimeric IL4/13 receptors that trigger the JAK-STAT signaling pathway mediated by STAT6, and signal-inhibitory phosphatase, CD43ex-45int (chimeric protein of the extracellular domain of CD43 and intracellular domain of CD45). In the absence of a target cell, CD43ex-45int can suppress downstream JAK-STAT signaling, but when the sensor cell binds to a target cell, CD43ex-45int is segregated from the cell–cell interface due to the physical force applied to its large extracellular domain, and becomes unable to suppress downstream signaling, so that transgene expression is triggered. One of the advantages of this system is that it is functional in nonimmune cells, while CAR function is not portable to non-immune cells (we note that SynNotch also works in nonimmune cells). Leveraging this feature, we engineered tumor-tropic mesenchymal stem cells with this contact-sensing system to drive expression of a cell-penetrating enzyme that can convert an anti-cancer prodrug into active form, enabling target-cell-specific enzyme-prodrug therapy. This work paves the way to achieve cancer therapy with engineered immune cells, and also for the first time shows that rational programming of dynamic movement of signaling proteins provides a new design principle to transmit extracellular information into cells.

We also showed that this kind of physical movement of signaling proteins upon specific cell contact can be used to programme a totally different function; target-specific synthetic cell invasion [43] (Fig. 4b). In nature, a living cell can invade another living cell in some contexts, forming a cell-in-cell structure (known as entosis, or emperipolesis). During this process, polarization of RhoA activity occurs in invader cells—specifically, low RhoA activity at the cell–cell interface and high RhoA activity at the rear side [44, 45]. We hypothesized this RhoA polarization is a sufficient condition for synthetically causing cell invasion, and we programmed the movement of RhoA by careful engineering of membrane proteins. Specifically, we switched the intracellular domain of the above-mentioned CD43ex-45int to constitutively active RhoA (RhoA_CA_) (or a RhoGEF, activator of RhoA), and conjugated dominant-negative RhoA (RhoA_DN_) to an antigen-binding receptor. In this setting, RhoA polarization does not occur in the absence of a target cell, but once the engineered cell binds to a target cell, RhoA_CA_ bound to CD43ex is segregated from the cell–cell interface by physical force, while RhoA_DN_ bound to an antigen binder accumulates at the interface, mimicking the RhoA polarization that occurs during the cell invasion process. We indeed found this RhoA polarization is a sufficient condition to cause cell invasion in our setting and succeeded in making target-cell-specific invader cells. By expressing a fusogenic protein that promotes membrane fusion in an acidic environment, we also succeeded in releasing the whole intracellular contents of the invader into the cytosol of receiver cells, enabling delivery of functional transcription factor, as well as fusion-driven cell ablation.

These findings demonstrate that careful programming of dynamic movement of membrane proteins upon specific cell contact in the sensor cells can be utilized to construct various cellular functions.

## Discussion

In this review, we have introduced state-of-art engineering principles that are available to make mammalian cells serve as “doctors” in the body, capable of sensing extracellular cues and exerting therapeutic functions in response. As CAR-T cells have already come onto the market, it is reasonable to consider that more and more types of engineered theranostic cells will be introduced next-generation disease therapies in the future.

We note that there are also other types of theranostic agents utilizing different biological sentinel systems, including viruses and bacteria. For example, viruses can be engineered to become capable of replicating only in cancer cells (oncolytic viruses) by deleting factors that are essential for viral replication in normal cells but not cancer cells (such as thymidine kinase [46–48]), or to become capable of exerting their functions only in target cells by introducing cell-specific promoters [49, 50, 50]. Certain types of bacteria can also be directed to accumulate at cancer sites by utilizing their anaerobic or chemotactic properties [51, 52] or by engineering their quorum-sensing properties [53]. Theranostic bacteria against metabolic disorders [54], colitis [55], and infection [56] have also been developed by engineering transcription-factor based input/output devices. These systems are outside the scope of the present review (for details of these systems, see elsewhere [2, 57–60]), but we consider that these systems and mammalian-cell-based theranostic systems are not competitive with each other, but rather are complementary. However, we would like to note that a significant advantage of using mammalian-cell-based systems is the availability of a much larger variety of biomolecular components (membrane receptors, signaling molecules, etc.) that can be used to sense input molecules, thereby expanding the spectrum of detectable disease markers, whereas viral and bacterial systems are usually dependent on transcription-factor-based switching that can sense only the presence or absence of small molecules. Also, mammalian-cell-based systems are expected to be inherently safer than virus/bacteria-based systems due to their lower immunogenicity and higher compatibility with the human body (i.e., they are made from mammalian cells).

Nevertheless, a key issue that remains for practical applications of engineered mammalian cells is adequate control quality of the engineered cells to meet the very high standards required for clinical use (in general, it is more difficult to engineer mammalian cells than viruses or bacteria due to their complexity). For CAR-T cells, only a single component is currently transduced in the cells. However, when researchers try to introduce complex, higher-order functions into cells, multiple components are likely to be involved. Since it is extremely difficult to transduce cells with multiple components in defined stoichiometry, development of single expression cassettes that can express multiple proteins in the appropriate ratio would be necessary. Alternatively, it might be of help to divide a complex program among different cell populations that communicate with each other, because the number of components in each cell can be reduced with this approach [61].

Further, precise control of the genome integration site is also very important. Currently, commercially available CAR-T cells are constructed by transduction using lentivirus or retrovirus, but it is difficult to control the integration site with the current system, and so there is a possibility of unpredictable side effects. To address this, Eyquem et al. showed that targeting CAR to the TRAC locus with the CRISPR/Cas9 system was useful [62]. Importantly, they not only achieved uniform CAR expression in human peripheral blood T cells with CRISPR-Cas9-based genome editing, but also showed that this enhances the T-cell potency, with the edited cells vastly outperforming conventional CAR-T. Thus, application of state-of-art gene editing technologies to construct engineered mammalian cells might be critical to deliver high functionality, reproducibility, and stability of engineered theranostic cells.

In order to expand the variety and improve the quality of soluble protein sensors, a new design principle that does not rely on protein dimerization seems desirable. Currently, to sense a protein that does not function as a homodimer, two different sensor proteins that bind to different epitopes on a single protein are required. It is often not easy to find multiple binders that bind to different, appropriate epitopes, and optimizing the combination and structures of two different receptor parts is also laborious. Further advances in receptor engineering technologies might help to address this issue.

To extend the theranostic usefulness of engineered mammalian cells, speeding up the response time of the system is also an important consideration for treating some types of diseases. To date, output expression mostly relies on transcriptional control of transgenes, which results in a slow (hours to days) response to stimuli [63]. However, for example, this time scale is not ideal for controlling blood insulin level, which should be precisely controlled depending on the rapidly changing blood sugar level. A new system to connect extracellular cues and output function in a rapid manner is needed.

Expanding the repertoire of output functions is also important. So far, output secreted from engineered cells that have sensed a disease state is mostly limited to effector proteins that modulate some target biological processes, but, for example, programming engineered cells to secrete highly sensitive reporter proteins would be beneficial for early diagnosis. Tastanova et al. showed that engineered cells capable of expressing tyrosinase, which produces a black pigment melanin from intracellular tyrosine upon sensing hypercalcemia, can function as a “biomedical tattoo” to detect hypercalcemia-associated cancers [64]. Coloration of the implant might allow patients to suspect cancer even at a very early stage, which would be beneficial to start treatment as early as possible. In addition, Aalipour et al. engineered macrophages to secrete Gaussia luciferase as a synthetic reporter upon recognition of cancer; this enables sensitive cancer diagnosis (they could detect tumors as small as 25–50 mm^3^) by measuring the blood luminescence signal) [65]. They cloned Gaussia luciferase under an arginase-1 promoter, whose expression seems to be activated by tumor-derived cytokines and metabolic intermediates. So far, this kind of system reports the presence of only a single biomarker, which limits the specificity of detection, but introduction of the logic gates described above might be a useful way forward.

Further, expanding the repertoire of output from proteins to other modalities would be also useful. For example, we reported implantable mammalian cells capable of secreting designer exosomes that can efficiently deliver encapsulated therapeutic mRNA into target cells [66]. By combining this kind of system to deliver functional nucleic acids and the sensor systems described in this review, it should be possible to construct on-demand RNA delivery systems.

As a different approach to extend the scope of diseases treatable with engineered cells, engineered mammalian cells can be used as a man–machine interface to realize highly personalized treatment. Folcher et al. constructed mammalian cells capable of secreting output proteins upon sensing near-infrared (NIR) light, and combined them with a brain–computer interface that monitors brain waves and enables a wireless-powered optogenetic implant to shine NIR light on implanted mammalian cells [67]. With this kind of design, it becomes possible to switch on secretion of therapeutic proteins when patients think they are sick. Similarly, Ye et al. developed engineered mammalian cells that can secrete therapeutic insulin or GLP-1 upon illumination with far-red light, and combined them with a red-light-emitting LED (light-emitting diode) operated by a smart-phone program [68]. With this kind of device, patients can remotely control their dose of therapeutic proteins on demand.

Apart from the function of such engineered cells, an important issue is the high cost of these kinds of cell-based medicine, which is a consequence of the need for expensive, high-end biotech instruments and laborious quality control procedures. A possible approach to reduce the cost of such therapies might be in situ cellular programming in vivo. Indeed, pioneering work has been done on in vivo generation of CAR-T cells by injection of functional nanoparticles [69]. Expanding the repertoire of in situ production approaches might greatly extend the scope of cell-based disease therapies in the future.

In this review, we have introduced state-of-art technologies to engineer theranostic mammalian cells and also have discussed future challenges that must be faced to make these emerging entities work effectively as “doctors” in the body. We believe current rapid advances in synthetic biology and related research areas will provide new strategies to overcome the various difficulties, given the enormous advantages that can be expected from fully personalized medicine.
